# Long-term exposure to PM_2.5_ and mortality in a national cohort in South Korea: effect modification by community deprivation, medical infrastructure, and greenness

**DOI:** 10.1186/s12889-024-18752-y

**Published:** 2024-05-08

**Authors:** Garam Byun, Sera Kim, Yongsoo Choi, Ayoung Kim, AiMS-CREATE Team, Jong-Tae Lee, Michelle L. Bell

**Affiliations:** 1 School of the Environment, Yale University, New Haven, CT 06511 USA; 2 Interdisciplinary Program in Precision Public Health, Department of Public Health Sciences, Graduate School of Korea University, Seoul, 02841 Republic of Korea; 3 Department of Public Health Sciences, Graduate School of Public health, Seoul National University, 1 Gwanak-ro, Gwanak-gu, Seoul, 08826 Republic of Korea; 4 Ai-Machine learning Statistics Collaborative Research Ensemble for Air pollution, Temperature, and all types of Environmental exposures, Seoul National University and Pusan National University, Seoul, Republic of Korea; 5 School of Health Policy and Management, College of Health Sciences, Korea University, Hana Science Hall, 145, Anam-ro, Seongbuk-gu, Seoul, 02841 Republic of Korea

**Keywords:** PM_2.5_, Mortality, Cohort study, Deprivation index, Medical infrastructure, Greenness

## Abstract

**Background:**

Long-term exposure to PM_2.5_ has been linked to increased mortality risk. However, limited studies have examined the potential modifying effect of community-level characteristics on this association, particularly in Asian contexts. This study aimed to estimate the effects of long-term exposure to PM_2.5_ on mortality in South Korea and to examine whether community-level deprivation, medical infrastructure, and greenness modify these associations.

**Methods:**

We conducted a nationwide cohort study using the National Health Insurance Service-National Sample Cohort. A total of 394,701 participants aged 30 years or older in 2006 were followed until 2019. Based on modelled PM_2.5_ concentrations, 1 to 3-year and 5-year moving averages of PM_2.5_ concentrations were assigned to each participant at the district level. Time-varying Cox proportional-hazards models were used to estimate the association between PM_2.5_ and non-accidental, circulatory, and respiratory mortality. We further conducted stratified analysis by community-level deprivation index, medical index, and normalized difference vegetation index to represent greenness.

**Results:**

PM_2.5_ exposure, based on 5-year moving averages, was positively associated with non-accidental (Hazard ratio, HR: 1.10, 95% Confidence Interval, CI: 1.01, 1.20, per 10 µg/m^3^ increase) and circulatory mortality (HR: 1.22, 95% CI: 1.01, 1.47). The 1-year moving average of PM_2.5_ was associated with respiratory mortality (HR: 1.33, 95% CI: 1.05, 1.67). We observed higher associations between PM_2.5_ and mortality in communities with higher deprivation and limited medical infrastructure. Communities with higher greenness showed lower risk for circulatory mortality but higher risk for respiratory mortality in association with PM_2.5_.

**Conclusions:**

Our study found mortality effects of long-term PM_2.5_ exposure and underlined the role of community-level factors in modifying these association. These findings highlight the importance of considering socio-environmental contexts in the design of air quality policies to reduce health disparities and enhance overall public health outcomes.

**Supplementary Information:**

The online version contains supplementary material available at 10.1186/s12889-024-18752-y.

## Introduction

Numerous epidemiological studies have established the link between long-term exposure to PM_2.5_ (particulate matter with aerodynamic diameter ≤ 2.5 μm) and premature mortality [1–5]. The Global Burden of Disease (GBD) Project estimated that ambient particulate matter pollution was responsible for 4.1 million deaths worldwide, including 21,837 in South Korea, in 2019 [6]. The health effects of PM_2.5_, however, is not uniform across individuals or groups. Some subpopulations are more vulnerable to the health effects of PM_2.5_ exposure, thus the health burden of PM_2.5_ is unevenly distributed, creating issues of environmental justice. Therefore, reducing health disparities from PM_2.5_ requires identification of the risk factors contributing to these disparities. Previous studies have reported that the PM-mortality associations could differ by individual sex, age, or socioeconomic status (SES) such as education level [7–10].

The health effects of PM_2.5_ could also be modified by community-level factors, which encompass complex, interconnected social, cultural, environmental, and economic systems that shape our environment and influence population health. For example, socio-economically deprived communities may face challenges in accessing information about air quality or adopting preventive measures, making them more susceptible to the harmful effects of PM_2.5_ [11]. Communities with better access to healthcare may be able to mitigate the health effects of air pollution more effectively than those with limited access [12]. Additionally, communities with more green spaces may provide residents with healthier environments, increasing social cohesion, physical activity, and overall well-being, thereby reducing the overall impact of PM_2.5_ exposure [13]. By targeting these modifiable factors at the community level, communities and policymakers could implement systemic changes that benefit entire populations, in addition to efforts that address individual behaviors or circumstances that may be more challenging to address.

Despite their importance, the role of community-level characteristics in modifying the effects of long-term PM_2.5_ exposure on mortality remains relatively underexplored. Some studies have investigated effect modification by community-level SES and found higher association between PM_2.5_ and mortality in communities with low SES levels [7, 14, 15]. However, these studies used limited indicators for community-level SES, such as education and income, which are useful but do not fully capture community-level SES, which has complex, multi-dimensional aspects. The authors suggested that the underlying mechanism by which community-level SES modifies the PM_2.5_-mortality association could be through limited access to health care. Nonetheless, there is a paucity of research investigating the role of health care accessibility in the health effects of PM_2.5_. A recent systematic review highlighted that only a small number of studies have examined role of greenness as an effect modifier in air pollution-health associations, and the results among these studies were not consistent [13]. A study conducted in South Korea examined modifying effects of community-level variables, including deprivation index, medical index, and greenness, on the relationship between long-term air pollution exposure and cardiovascular mortality, but did not find significant differences in effect estimates [16]. Their study, however, was restricted to seven cities, and only focused on PM_10_ (particulate matter with aerodynamic diameter ≤ 10 μm), whereas our nationwide study analyzed PM_2.5_. While both forms of particulate matter are harmful to human health, PM_2.5_ is more detrimental as it penetrates deeper into the respiratory system [17].

In this study, we estimated the effects of long-term exposure to PM_2.5_ on all-cause and cause-specific mortality in South Korea using a nation-wide population-based cohort. We also examined whether these associations are modified by community-level characteristics including deprivation, medical infrastructure, and greenness.

## Materials and methods

### Study population

We obtained National Sample Cohort from the National Health Insurance Service (NHIS-NSC, version 2.2), which is a population-based cohort consisting of approximately 1 million samples, representing 2% of the total Korean population. The baseline population was sampled in 2006, with a bidirectional follow-up from 2002 to 2019. The data included participants’ socio-demographic information and district-level residential addresses (referred to as si-gun-gu in South Korea, which is roughly analogous to the US city), which were updated annually. The data also provided health examination information, such as smoking status, frequency of alcohol use, physical activity, and body mass index (BMI), for a subpopulation who participated in the examination. The details of the cohort profiles have been previously described [18].

The study population was limited to those who were not living in Jeju Island in the baseline year, 2006. We excluded residents in Jeju since exposure modeling data were unavailable for the island area. Out of 1,021,208 individuals enrolled in 2006, 11,527 were living in Jeju. We further restricted the study population to those aged 30 years and over in 2006, with no missing residential information between 2002 and 2005, and who underwent the health examinations at least once during 2006–2015 without missing information on smoking status, alcohol use, physical activity, or BMI. The final number of participants included in the analysis was 394,701 (Figure S1).

We determined the occurrence of subjects’ deaths using death records linked to the NHIS-NSC data. We used the 10th Revision of the International Classification of Diseases (ICD-10) to define all-cause deaths excluding accidental causes (ICD-10, A99-R99), and deaths from circulatory diseases (ICD-10, I00-I99), and respiratory diseases (ICD-10, J00-J99).

### Exposure assessment

Ambient PM_2.5_ concentrations were estimated by AiMS-CREATE (Ai-Machine learning and Statistics Collaborative Research Ensemble for Air pollution, Temperature, and all types of Environmental exposure) team. The developed ensemble model combined three machine learning models—random forest, light gradient boosting, and neural network—to estimate monthly PM_2.5_ concentrations at a spatial resolution of 1 × 1 km² across 226 districts in South Korea from 2002 to 2020. The model utilized a comprehensive set of predictors, including atmospheric, meteorologic, and land-use variables derived from satellite-based data via the Google Earth Engine and Socioeconomic Data and Applications Center, inverse distance weighted ground-level air pollutants concentrations calculated with national monitoring data, and regional socioeconomic variables from a database of community health outcomes and health determinants provided by the Korean Disease Control and Prevention Agency. The ensemble PM_2.5_ model demonstrated a cross-validated R² of 0.87. More detailed information on the PM_2.5_ modeling method are described previously [19]. Based on these data, we calculated district-level annual mean concentrations of PM_2.5_ and assigned them to individuals based on their residential addresses for each calendar year of follow-up from 2002 to 2019. Consequently, for each year, individuals residing in the same district received the same PM_2.5_ concentration assignment. Taking into account the downward trend in PM_2.5_ concentrations over the years in South Korea, we treated PM_2.5_ exposure as a time-varying variable. Finally, long-term exposures for each individual were defined as 1-year, 2-year, 3-year, and 5-year moving averages of the assigned PM_2.5_ concentrations. The 4-year moving average was omitted due to preliminary findings indicating marginal differences between the 4-year and 5-year averages, thus simplifying the analysis and focusing on the most relevant exposure durations. For instance, if an individual was followed from 2006 to 2010, the 1-year moving average exposures would be assigned PM_2.5_ concentrations for 2006, 2007, and so on, up to 2010; the 5-year moving average exposures would be the mean of PM_2.5_ concentrations assigned over 2002–2006, 2003–2007, and so forth, up to 2006–2010. To assess the correlation between different time windows of PM_2.5_ exposure, Pearson correlation coefficients were calculated for each pair of time windows. Pearson correlation was chosen over Spearman correlation based on the assumption of a linear relationship and normal distribution of data. In the following analysis that estimated the association between PM_2.5_ and mortality, the exposure time window exhibiting the highest association with mortality were selected as the main exposures for each cause of death.

### Community-level variables

Data on indicators for the deprivation index were obtained from 2005, 2010, 2015, and 2020 population and housing census data at the district level. The deprivation index was calculated by standardizing and summing the following eight indicators: proportion of households without a car, proportion of households below the minimum housing standard, proportion of single-person households, proportion of households with a female household head, proportion of households not living in an apartment, proportion of people aged 65 years or over, proportions of people without a high school diploma among those aged 30–64 years, and proportion of divorced or widowed individuals among those aged 15 years or over [20]. Deprivation indexes for 2002–2004, 2006–2009, 2011–2014, and 2016–2019 were imputed with data of 2005, 2010, 2015, and 2020, respectively.

Data on indicators for the medical index were obtained from the Korean Statistical Information Service at the district level, annually spanning 2002 to 2019. The medical index was calculated by standardizing and summing the numbers of medical personnel, hospitals, and hospital beds per capita. These three indicators are commonly used to represent medical resources in South Korea [21], and several previous studies have adopted the medical index as a summary measure of these indicators [16, 22–25]. Table S1 in the supplementary material provides the detailed methods used to calculate the variables for the deprivation and medical indices.

As a measure of residential greenness or greenspace, we utilized the normalized difference vegetation index (NDVI). NDVI data, with a 250-meter spatial resolution and collected at 16-day intervals from 2002 to 2019, yielding approximately 23 observations per year, were obtained from the moderate resolution imaging spectroradiometer (MODIS) of the National Aeronautics and Space Administration (NASA) Terra satellite (MOD13Q1 product). All non-negative NDVI values of pixels within the boundaries for each district were averaged. Annual representative NDVI values were determined as the median values for each year between May and October. The selection of NDVI values for this specific season aims to reflect the peak greenness period in South Korea. The choice of median NDVI, rather than the maximum value, is due to the potential for measurement errors in NDVI data, making the median a more reliable indicator of typical vegetation conditions. All community-level variables were treated as time-varying variables matching the PM_2.5_ exposure definition. The correlations between each community-level variable and PM_2.5_ exposure were analyzed, along with the correlations among the community-level variables themselves. Since the community-level variables exhibited minimal variation across different time windows, we performed these correlation analyses using only one of our main exposure time windows, the 5-year moving average.

### Statistical analyses

We employed the time-varying Cox-proportional hazards model to estimate the association between PM_2.5_ exposure and mortality, using calendar year as the time scale. Subjects were considered censored when they moved out of the study region (e.g., to Jeju Island or out of the country), at the time of lost-to-follow-up, or at the end of the study period, if death was not confirmed. In the analyses for cause-specific mortalities, individuals who died from other causes were treated as censored at the year of death. The baseline hazard was stratified by sex and age (10-year intervals), and each model was adjusted for income (measured as insurance premium; categorized into zero to second decile, third to fifth decile, sixth to eighth decile, and nineth to tenth decile), smoking (never, former, and current), alcohol drinking frequency (≤ once a month, 2–3 times a month, and ≥ once in a week), physical activity (< once a week and ≥ once a week), BMI (underweight, < 18.5; normal, 18.5 ≤ BMI < 23, overweight, 23 ≤ BMI < 25; and obese, ≥ 25 kg/m^2^), deprivation index (categorized into tertiles), medical index (categorized into tertiles), NDVI (categorized into tertiles), and indicator for residential province at baseline (the study area consisted of 16 provinces). The categorizations of these covariates were predetermined based on previous studies utilizing the NHIS-NSC dataset [23, 26]. Among individual-level covariates, we used baseline information for sex, age, and income, and used the first health examination information of each individual from 2006 to 2019 for the remaining covariates. Long-term PM_2.5_ exposure and community-level covariates were treated as time-varying variables. Hazard ratios (HRs) and 95% confidence intervals (CIs) were presented in relation to 10 µg/m^3^ increase in PM_2.5_. The proportional hazard assumption of the model was tested using the scaled Schoenfeld residuals, confirming the validity of the assumption (*p*-value > 0.05).

For sensitivity analyses, we included additional adjustments for nitrogen dioxide (NO_2_), ozone (O_3_), and temperature in the model one at a time. Modeling data for NO_2_ and O_3_ were also estimated by AiMS-CREATE team [27], and ERA5-land satellite temperature data were obtained through Google Earth Engine [19]. We defined the exposure to NO_2_, O_3_, and temperature in the same method as PM_2.5_, using district-level annual averages. The main model assumed a linear relationship between PM_2.5_ and mortality. To examine the non-linear relationship between PM_2.5_ and mortality, we fitted a spline function of PM_2.5_ instead of a linear term in the main model. A natural cubic spline with three degrees of freedom was used, based on the methodology of previous studies in South Korea that investigated the non-linear association between long-term air pollution exposure and mortality [16, 23].

To assess whether community-level variables modify the association between PM_2.5_ and mortality, we conducted stratified analyses by each community-level variable. Additionally, we included a multiplicative interaction term between PM_2.5_ and each community-level variable in the model, one at a time. The statistical significance of the effect modification was determined based on the *p*-values of the interaction terms. Given the potential low statistical power of the interaction test [28], significance levels of 0.1 and 0.2 were considered alongside the conventional 0.05 threshold to interpret the interaction terms. When evaluating a specific community-level variable as an effect modifier, the other community-level variables were included in the model as covariates.

The analyses were performed with SAS software, version 9.4 (SAS Institute, Cary, North Carolina, United States) and R software, version 3.3.3 (R Foundation for Statistical Computing, Vienna, Austria). The R packages “sf” and “rgdal” were used to aggregate gridded PM_2.5_ data into district-level data, and “survival” and “survminer” were used for Cox regression analysis.

## Results

### Descriptive statistics

A total of 394,701 participants were included in 2006 and followed up until 2019. The average follow-up time was 12.6 years. The characteristics of the study population are presented in Table 1. Males comprised 50.3% of the population, and 20.0% were aged 60 years or older. More than half of the population never smoked, drank alcohol once a month or less, were overweight or obese, and engaged in physical activity at least once a week. During the follow-up period, there were 25,812 non-accidental deaths, of which 5,916 and 2,814 were due to circulatory and respiratory diseases, respectively.

**Table 1:** Population characteristics at baseline and the number of deaths

Variables	Number	(%)
Total	394 701	(100.0)
Sex		
Male	198 697	(50.3)
Female	196 004	(49.7)
Age (years)		
30–39	107 177	(27.2)
40–49	121 884	(30.9)
50–59	86 608	(21.9)
60–69	51 640	(13.1)
70–79	23 873	(6.0)
≥ 80	3 519	(0.9)
Level of income		
0-2nd decile	57 762	(14.6)
3rd-5th decile	99 475	(25.2)
6th-8th decile	130 011	(32.9)
9th-10th decile	107 453	(27.2)
Smoking status		
Never	260 713	(66.1)
Former	42 273	(10.7)
Current	91 715	(23.2)
Alcohol drinking frequency		
≤ Once a month	222 979	(56.5)
2–3 times a month	64 254	(16.3)
≥ Once in a week	107 468	(27.2)
Body mass index (kg/m^2^)		
< 18.5	11 717	(3.0)
18.5 ≤ BMI < 23	146 220	(37.0)
23 ≤ BMI < 25	98 884	(25.1)
≥ 25	137 880	(34.9)
Physical activity		
< Once a week	179 427	(45.5)
≥ Once a week	215 274	(54.5)
Causes of deaths		
Non-accidental	25 812	(6.5)
Circulatory disease	5 916	(1.5)
Respiratory disease	2 814	(0.7)

**Fig. 1:**
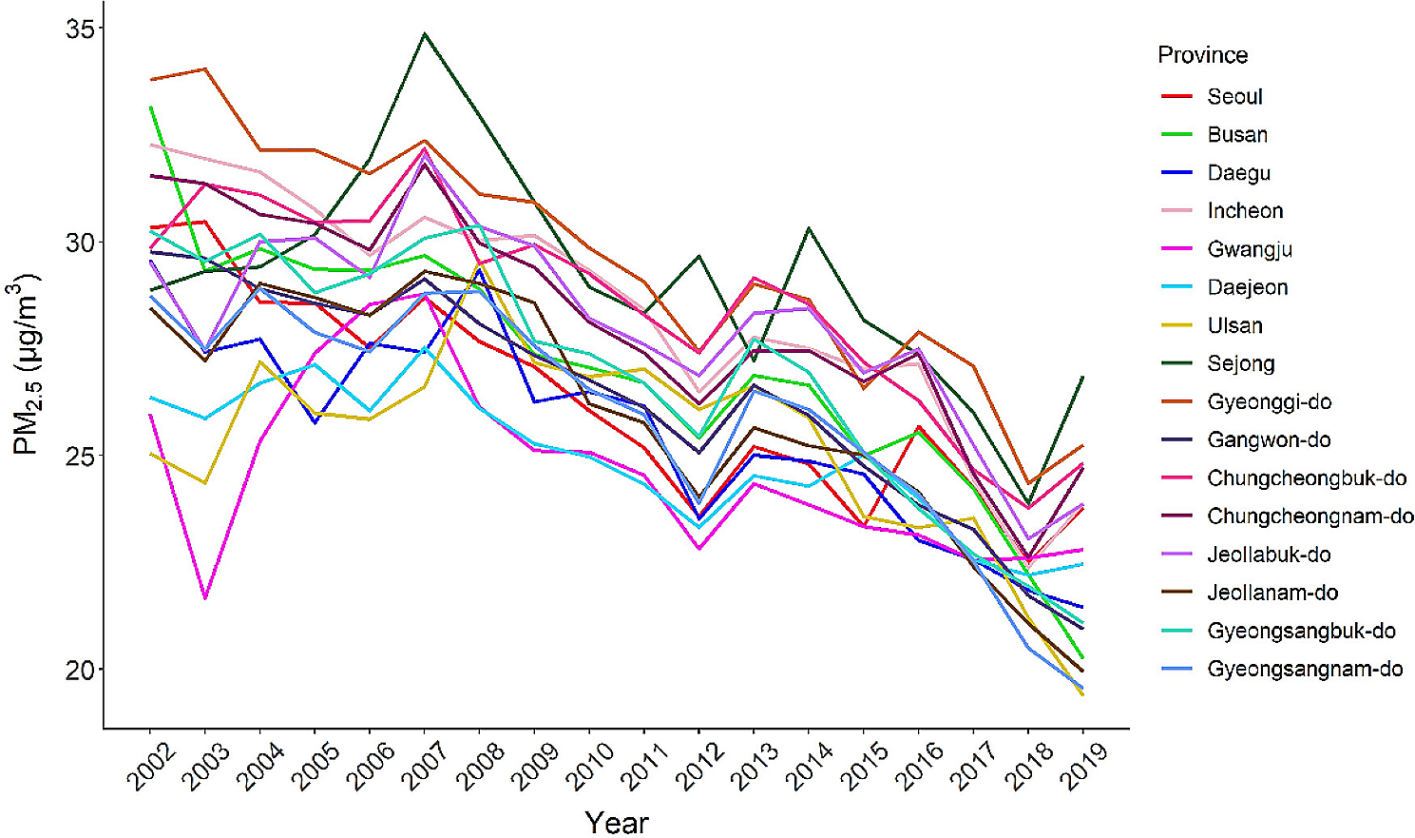
Temporal trends in annual average PM_2.5_ concentrations for 16 provinces in South Korea (2002–2019)

Figure 1 shows the annual average PM_2.5_ concentrations from 2002 to 2019 for the 16 provinces in South Korea. Generally, there has been a decreasing trend in PM_2.5_ levels over the years in most provinces. As of 2019, Sejong reported the highest average PM_2.5_ concentration (26.8 µg/m^3^), while Ulsan had the lowest (19.3 µg/m^3^). The district-level distribution of PM_2.5_ concentration is given in the Additional file 1 (Figure S2). Additionally, maps of key geospatial variables can be found in Figure S3. The mean population exposure to 5-year moving averages of PM_2.5_ was 27.29 µg/m^3^ (standard deviation, SD 2.61), which exceeds the annual PM_2.5_ air quality guideline of 15 µg/m^3^ in South Korea (Table S2). The 5-year moving averages (SD) of participants’ residential deprivation index, medical index, and NDVI were − 3.80 (4.97), 0.13 (1.95), and 0.56 (0.13), respectively (Table S2). Different PM_2.5_ exposure time windows were highly correlated (*r* ≥ 0.9), and the PM_2.5_ exposure and community-level variables exhibited weak correlations (*r* ≤ 0.2) (Table S3).

### Associations between PM_2.5_ exposure and mortality

The associations between different long-term PM_2.5_ exposure time windows and mortality are presented in Table 2. The HRs for non-accidental mortality were similar for 2, 3, and 5-year moving average exposures to PM_2.5_ (HR: 1.10, 95% CI: 1.01, 1.20, for 10 µg/m^3^ increase). The highest HR for circulatory mortality was observed in the 5-year moving average PM_2.5_ (HR: 1.22, 95% CI: 1.01, 1.47), while the highest HR for respiratory mortality was found in the 1-year moving average PM_2.5_ (HR: 1.33, 95% CI: 1.05, 1.67). The main exposures were selected as the 5-year moving average PM_2.5_ for non-accidental and circulatory mortality, and the 1-year moving average PM_2.5_ for respiratory mortality. Subsequent analyses were conducted using only the selected main exposures for each outcome. Sensitivity analyses that included adjustments for NO_2_, O_3_, or temperature did not result in substantial changes to the estimates (Table S4).

**Table 2:** Hazard Ratios (HRs) and 95% Confidence Intervals (CIs) of mortality associated with 10 µg/m^3^ increase in long-term exposure to PM_2.5_.*

Cause of death	Exposure time window	HR (95% CI)
Non-accidental	1-year moving average	1.08 (1.00, 1.17)
	2-year moving average	1.10 (1.01, 1.20)
	3-year moving average	1.10 (1.01, 1.20)
	5-year moving average	1.10 (1.01, 1.20)
Circulatory disease	1-year moving average	1.09 (0.93, 1.28)
	2-year moving average	1.18 (0.99, 1.40)
	3-year moving average	1.16 (0.97, 1.39)
	5-year moving average	1.22 (1.01, 1.47)
Respiratory disease	1-year moving average	1.33 (1.05, 1.67)
	2-year moving average	1.32 (1.02, 1.70)
	3-year moving average	1.29 (0.99, 1.68)
	5-year moving average	1.27 (0.96, 1.67)

*Stratified by age and sex, and adjusted for income, smoking, alcohol use, physical activity, body mass index, medical index, deprivation index, normalized difference vegetation index, and indicator for province

**Fig. 2:**
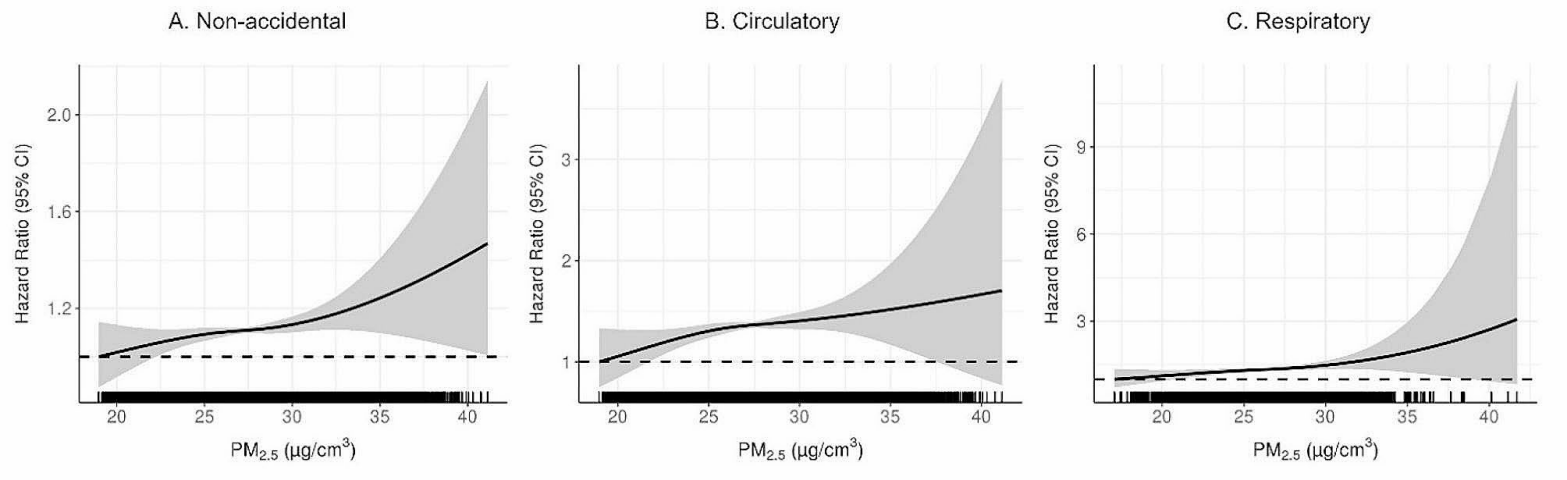
Exposure-response relationship between PM_2.5_ and mortality due to non-accidental causes (**A**), circulatory diseases (**B**), and respiratory diseases (**C**) *Note*. The solid line represents the hazard ratio, and the shaded area represents the 95% CI. HR refers to risk for a 10 µg/m^3^ increase in PM_2.5_

Figure 2 displays the exposure-response relationships between PM_2.5_ and mortality. Exposure time windows were set as a 5-year moving average for non-accidental and circulatory mortality, and a 1-year moving average was applied for respiratory mortality. For all three outcomes, the exposure-response curve exhibits a near-linear relationship, with a monotonic increase in mortality risk as PM_2.5_ exposure increases. The curve also indicated no threshold in the relationship, suggesting that adverse health effects from PM_2.5_ exposure may occur even at low levels, although estimates in low exposures were statistically uncertain, possibly due to the small sample size for those levels of exposure. The shape of the exposure-response relationships at high exposure levels, above approximately 35 µg/m^3^, exhibited uncertainty with wide CIs due to limited data points.

### Effect modification by community-level characteristics

**Fig. 3:**
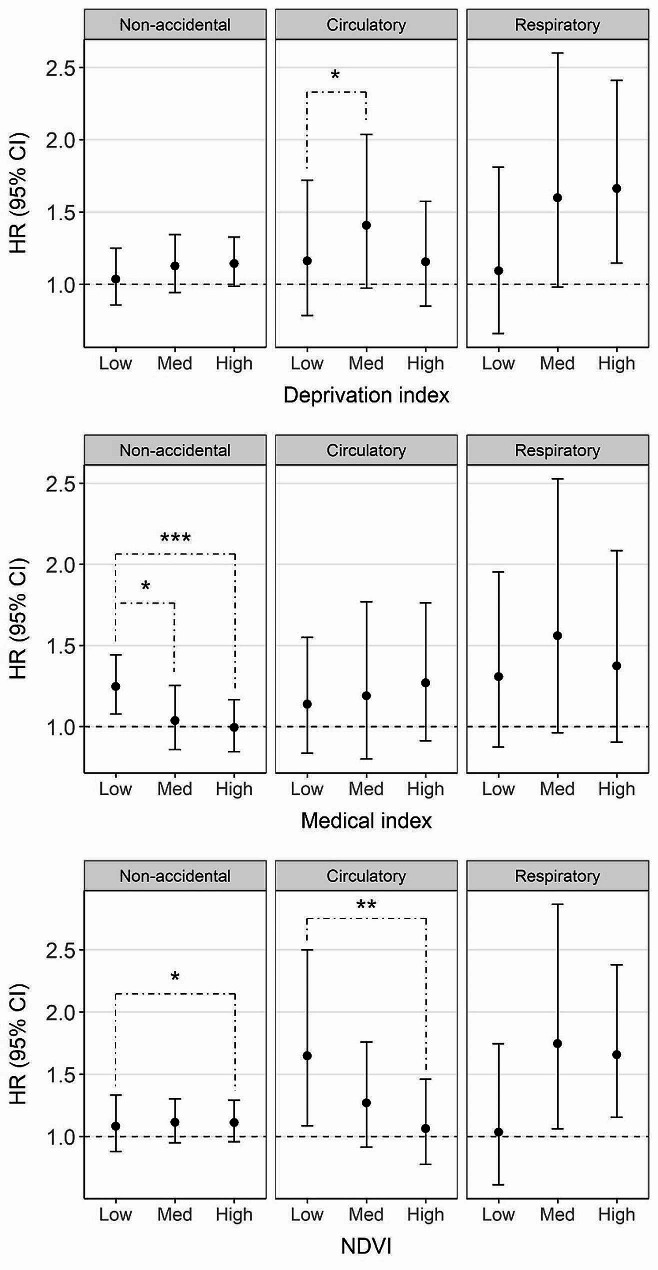
Associations between PM_2.5_ and mortality stratified by community-level variables, deprivation index, medical index, and NDVI (normalized difference vegetation index) *Note*. ‘Low’, ‘Med’, and ‘High’ represent the 1st, 2nd, and 3rd tertile of each community-level variable, respectively. The black dot represents the hazard ratio (HR), and the solid vertical line represents the 95% CI per 10 µg/m^3^ increase in PM_2.5_. Single, double, and triple asterisks indicate that the *p*-value for the interaction term is significant at the 0.2, 0.1, and 0.05 levels, respectively

Figure 3 presents the associations between PM_2.5_ and mortality across different community-level characteristics. The numerical effect estimates for Fig. 3 can be found in the Additional file 1 (Table S5). For all three outcomes, the lowest associations were observed in the least deprived communities (low deprivation index), while higher associations were found in more deprived communities (medium or high deprivation index). Statistically significant (at 0.2 level) differences in the associations between PM_2.5_ and mortality by medical index were observed only for non-accidental mortality, with the highest association in communities with limited medical infrastructure (low medical index). Contrasting patterns in the association between PM_2.5_ and mortality by NDVI were observed for circulatory and respiratory mortality; the lowest HR was found in communities with the most green space (high NDVI) for circulatory mortality, while the lowest HR was found in communities with the least green space (low NDVI) for respiratory mortality.

## Discussion

To the best of our knowledge, this is the first nationwide study to investigate the association between long-term exposure to PM_2.5_ and mortality in South Korea. In this study, we found that long-term exposure to PM_2.5_ was significantly associated with increased risk of non-accidental, circulatory, and respiratory mortalities. We further observed that community-level characteristics including deprivation status, medical infrastructure, and greenness could modify these associations, and the direction of this modification might not be consistent across different causes of death.

Our findings, which indicate a positive association between long-term PM_2.5_ exposure and mortality, are in line with the majority of the existing epidemiological evidence. A recent systematic review and meta-analysis reported a summary risk ratio of 1.08 (95% CI 1.06, 1.09), 1.11 (95% CI 1.09, 1.14), and 1.10 (95% CI 1.03, 1.18) per 10 µg/m^3^ increase in long-term PM_2.5_ exposure for all-cause, circulatory, and respiratory mortality, respectively [2]. However, most previous studies on long-term PM_2.5_ exposure and mortality have been conducted in American and European regions. The differences in observed effect sizes of PM_2.5_ in the present study compared to previous studies may have resulted from methodological differences, variations in PM_2.5_ concentration and composition, or distinct population characteristics.

In South Korea, only a handful of cohort-based studies have investigated the long-term exposure to PM_10_ and mortality. One nationwide study based on NHIS-NSC version 1.0 reported positive, but statistically non-significant associations between PM_10_ and mortality: HR (95% CI) of 1.05 (0.99, 1.11), 1.02 (0.90, 1.16), 1.19 (0.91, 1.57) per 10 µg/m^3^ increase in PM_10_, for non-accidental, cardiovascular, and respiratory mortality, respectively [29]. A study using Korean National Health and Nutritional Examination Survey with Mortality follow-up found positive association of PM_10_ with circulatory mortality (HR 1.27; 95% CI 0.96, 1.66 per 10 µg/m^3^ increase), but not with respiratory mortality, in seven major cities in South Korea [16]. To juxtapose our findings with previous studies in South Korea, we changed the exposure in our main model from PM_2.5_ to PM_10_. Consistent with earlier findings, we observed insignificant associations between PM_10_ and mortalities: HR (95% CI) of 0.96 (0.91, 1.02), 1.09 (0.97, 1.22), 1.11 (0.95, 1.31) per 10 µg/m^3^ increase in PM_10_ for non-accidental, cardiovascular, and respiratory mortality, respectively. These results suggest that exposure to PM_2.5_ might pose a greater risk to human health than PM_10_ in South Korea. PM_2.5_ is capable of reaching the alveolar region of the lungs where gas exchange occurs due to its finer size, and may contain more hazardous substances, including metallic components, than larger particles [17]. Further epidemiological studies focusing on PM_2.5_ would be warranted in South Korea.

In the present study, we observed that circulatory mortality exhibited a higher association with longer exposure time windows of PM_2.5_, while respiratory mortality was more highly associated with shorter exposure time windows of PM_2.5_. Although the reasons for these differential associations remain unclear, potential explanations might lie in the different underlying biological mechanisms. The respiratory system encounters direct and immediate exposure to ambient PM_2.5_, where inhaled particulates can trigger tissue damage, inflammation, and oxidative stress in the respiratory tract. On the other hand, the effects of PM_2.5_ on the circulatory system are likely more indirect. For example, inflammatory mediators like cytokines, generated in the respiratory tract due to PM_2.5_ exposure, can infiltrate the circulatory system and instigate distant pathophysiological changes potentially leading to pronounced cardiovascular disease [30–33]. Previous studies examining the lag structure of the association between short-term exposure to PM_10_ and mortality in South Korea reported that respiratory mortality was more affected by immediate exposure (e.g., same day), whereas effects on cardiovascular mortality were associated with more lagged and prolonged exposure [34, 35].

Regarding effect modification by community-level characteristics, we found higher effect of PM_2.5_ on mortality in more deprived community, although the differences in effect estimates were statistically insignificant overall. Individual- or community-level SES, which are commonly measured by education or income, have been reported as important modifiers on the PM-mortality relationship both in short-term and long-term effects [7–9, 14, 15]. In this study, we used the deprivation index as a more comprehensive measure of community-level SES, encapsulating multiple dimensions of deprivation, including income, education, marital status, and housing conditions. The indicators for this index were sourced from previous study by Choi et al., 2019, chosen specifically to represent local economic and social deprivation within the South Korean context. In light of this, our finding indicating elevated PM_2.5_-related mortality in more deprived communities may be understood as reflecting systemic deprivation in these areas, rather than merely individual health conditions or behaviors tied to individual SES. For instance, deprived communities could face structural disadvantages such as limited access to healthcare services, inadequate infrastructure, and substandard housing conditions, which could enhance their susceptibility to the health effects of PM_2.5_. Further, deprived communities may also have limited access to resources for adapting or responding to air pollution, such as air purifiers, air-conditioned spaces, and health information. Conversely, it’s possible that more deprived areas are less impacted by PM_2.5_ compared to less deprived areas. For instance, areas with higher levels of deprivation might be more rural, potentially leading to lower population density and fewer sources of anthropogenic air pollution. This could result in different levels or chemical composition of PM_2.5_ in these regions.

Insufficient healthcare accessibility has often been posited as a potential pathway through which low SES may modify the PM_2.5_-mortality relationship, but empirical investigations into this premise have been scarce. Our study showed that communities with limited medical resources were more vulnerable to the health effects of long-term PM_2.5_ exposure. Such regions may have a lower capacity to manage chronic health conditions that are exacerbated by long-term PM_2.5_ exposure or a lack of timely and appropriate medical care, exacerbating the impacts of PM_2.5_ on mortality. Indeed, studies have suggested an increased mortality risk associated with greater distances to hospitals [36, 37]. Additionally, these communities might have limited access to preventive health measures, such as health education and screening programs, which can mitigate the harmful effects of PM_2.5_ exposure. However, our findings also indicated a heightened effect of PM_2.5_ on cause-specific mortalities in areas with more medical resources, although the differences were not statistically significant. One possible explanation is that areas with more medical resources could have more advanced disease detection and reporting systems, potentially resulting in a higher number of documented mortality cases associated with PM_2.5_ exposure. Furthermore, areas with more medical resources might serve a population with a higher proportion of vulnerable individuals, such as the elderly or those with pre-existing health conditions, who might be seeking enhanced healthcare services. This population might be more susceptible to the effects of PM_2.5_.

Modifying effects of green spaces on the relationship between PM_2.5_ and mortality outcomes exhibited contrasting patterns for circulatory and respiratory causes in this study. We observed that communities with higher green space had a lower risk of circulatory mortality in association with PM_2.5_, but conversely, a higher risk of respiratory mortality. Akin to these findings, a study conducted in seven major cities in South Korea found higher cardiovascular mortality associated with PM_10_ in communities with a lower level of greenness, while the risk of PM_10_ on non-accidental mortality was higher in greener communities [38]. Several previous studies have suggested the beneficial effect of greenness in mitigating the health risk of PM exposure by fostering more opportunities for physical activity, enhancing social interactions, and reducing psychological stress levels [39–41]. However, green space can also potentially have adverse effects on health under certain conditions. The release of allergenic pollens during peak blooming times can trigger allergies and aggravate respiratory issues. The use of pesticides in maintaining these areas may result in direct exposure or contaminate the environment. Furthermore, green spaces can emit biogenic volatile organic compounds that contribute to the formation of ground-level ozone and secondary organic aerosols, potentially worsening air quality and respiratory health. Additionally, dense vegetation can sometimes harbor vectors of diseases, such as ticks and mosquitoes, leading to an increased risk of vector-borne diseases in greener areas [42, 43]. The influence of greenness on the association between PM_2.5_ and mortality could be attributed to a combination of these potential mechanisms, warranting further research to untangle their intricate interactions.

Our measures for community-level characteristics come with some limitations. First, we measured indicators for the deprivation index every five years, restricting our ability to capture temporal variations in community-level SES. In addition, we could not include employment status indicators due to the data availability. These shortcomings are unlikely to significantly impacted our results because the deprivation index did not show considerable variations over time and incorporated multiple dimensions of SES that are likely to correlate closely with employment. Second, while the medical index used in our study provides a general overview of the medical resources available within a given district, it might not accurately reflect the actual healthcare accessibility experienced by the residents. It incorporates the quantity of medical personnel, hospitals, and hospital beds per capita, but it does not take into account the distribution of these resources within the districts or the potential barriers that may hinder access to these resources, such as transportation difficulties, costs, or the quality of care provided. Third, NDVI as a measure of vegetation does not reflect the different types of vegetation that could impact health differently. For example, dense forests might have different health effects compared to grasslands or urban parks. NDVI might not adequately represent pollen concentrations and their potential health impacts, as it does not account for variations in flowering times and pollen release across different types of vegetation. Furthermore, NDVI does not account for the accessibility of green spaces or their quality, both of which are important aspects of greenness that can affect health outcomes. In this study, we examined the modifying effect of each community-level characteristic independently, rather than in conjunction. Community-level variables do not account for activity patterns of study participants that would affect exposure, such as sub-community heterogeneity or movement from the community of residence to different communities for work or school. In addition. In real-world contexts, various community-level factors, including those not considered in our study, could interact with each other and consequently influence the associations between PM_2.5_ and mortality. Although the correlations among the community-level factors investigated in our study were relatively weak, these factors could still exert combined effects. Thus, future research is called for, to more comprehensively understand health disparities associated with PM_2.5_ exposure for various communities.

This study also has potential exposure measurement errors. We used district-level PM_2.5_ concentrations since individual-level exposure measurements or individual residential addresses were not available. While we used exposure estimates for PM_2.5_ from a validated model, which brings the strength of higher spatial resolution than would exposures based on air pollution monitors, these exposures are estimates there are inherent uncertainties. Our estimates are based on ambient air pollution, which do not incorporate differences in exposure patterns due to indoor air pollution, indoor/outdoor activity patterns, or occupational exposures. The modeled ambient PM_2.5_ concentrations are also subject to errors arising from uncertainties in the input variables. Specifically, observed air pollution data are constrained by the coverage and density of the existing monitoring network, potentially not fully representing all areas. However, the prediction model for PM_2.5_ showed excellent performance (cross-validated R^2^: 0.87), providing national coverage across South Korea [19]. We also took into account the change in subjects’ residential locations throughout the follow-up period when assigning PM_2.5_ concentrations, which further reduces potential exposure misclassification.

Lastly, while the NHIS-NSC dataset offers a large and representative sample of the South Korean population through the universal insurance coverage system and systematic stratified random sampling, not all participants could be included in our analysis. Specifically, 204,213 participants were excluded due to missing information on covariates such as smoking status and BMI (Figure S1). The characteristics of this excluded group may differ from those of the study population, introducing the possibility of selection bias. Despite these limitations, this is the first cohort study to examine the mortality effects of long-term exposure to PM_2.5_ using the nationwide sample of South Korea, to the best of our knowledge. We also demonstrated the unequal effects of PM_2.5_ by community-level characteristics. Our findings suggest the necessity for policy strategies to extend beyond simple mitigation of PM_2.5_ levels and encompass local socio-environmental contexts. This integrated approach can help mitigate the health impacts of air pollution more effectively, promoting health equity among different communities.

## Conclusion

In conclusion, this nationwide population-based study found adverse effects of long-term exposure to PM_2.5_ on mortality in South Korea, with heightened risk in communities characterized by high deprivation and limited medical infrastructure. Additionally, greenness showed contrasting effects on circulatory and respiratory mortality associated with PM_2.5_, highlighting the multifaceted roles of community-level characteristics in shaping the health effects of air pollution. These findings emphasize the importance of integrating socio-economic and environmental factors into air quality policies to promote health equity across communities.

### Electronic supplementary material

Below is the link to the electronic supplementary material.

Additional File 1: Supplementary Material (.docx)

## Data Availability

The air pollution prediction data can be accessed upon request, subject to approval from the AiMS-CREATE team (https://www.datascience4health.com). As for the health data, NHIS-NSC 2.2, the authors are not permitted to distribute it directly; however, interested researchers can apply for the data through the NHIS (https://nhiss.nhis.or.kr). The community-level variables data are publicly available and can be accessed from the sources mentioned in the main text.

## Abbreviations

AiMS-CREATE: Ai-Machine learning and Statistics Collaborative Research Ensemble for Air pollution, Temperature, and all types of Environmental exposure
BMI: Body mass index
CI: Confidence interval
GBD: Global Burden of Disease
HR: Hazard ratio
ICD: International Classification of Diseases
NDVI: Normalized difference vegetation index
NO_2_: Nitrogen dioxide
O_3_: Ozone
PM_2.5_: Particulate matter with aerodynamic diameter ≤ 2.5 μm
SD: Standard deviation
SES: Socioeconomic status
