# Supplementary material for: Long-term exposure to PM2.5 and mortality in a national cohort in South Korea: effect modification by community deprivation, medical infrastructure, and greenness
Source: BMC Public Health. 2024 May 8;24:1266. doi: 10.1186/s12889-024-18752-y (PMC11080206; doi:10.1186/s12889-024-18752-y)
Supplement: Supplementary file 1 — Additional File 1: Supplementary Material (.docx) [file 12889_2024_18752_MOESM1_ESM.docx]

**Supplementary Material**

**Long-term exposure to PM_2.5_ and mortality in a national cohort in South Korea: Effect modification by community deprivation, medical infrastructure, and greenness**

Garam Byun, Sera Kim, Yongsoo Choi, Ayoung Kim, AiMS-CREATE Team, Jong-Tae Lee, Michelle L. Bell,

**Table of Contents**

**Table S1.** Calculation methods for district-level variables used in the deprivation index and medical index.

**Table S2.** Summary statistics of 5-year moving averages of PM_2.5_ exposure and community-level variables.

**Table S3.** Pearson's correlation coefficients between PM_2.5_ exposure and community level-variables.

**Table S4.** Hazard ratios (HRs) and 95% confidence intervals (CIs) of mortality associated with 10 μg/m^3^ increase in PM_2.5_ with additional adjustments.

**Table S5.** Hazard ratios (HRs) and 95% confidence intervals (CIs) of mortality associated with 10 μg/m^3^ increase in PM_2.5_, stratified by deprivation index, medical index, and NDVI (normalized difference vegetation index).

**Figure S1.** Flow chart of the selection process of the study participants

**Figure S2.** Map of predicted PM_2.5_ concentrations averaged at district level from 2002 to 2019 in South Korea

**Figure S3.** Maps of geospatial variables at the district level in South Korea.

**Table S1.** Calculation methods and data sources for district-level variables used in the deprivation index and medical index.

| Index | Source | Variable | Calculation method |
| --- | --- | --- | --- |
| Deprivation index | Korea Population and Housing Census via Korean Statistical Information Service | Proportion of households without a car | The number of households that do not own a car divided by the total number of households |
|  |  | Proportion of households below the minimum housing standard | The number of households satisfied with at least one of the following five divided by the total number of households: 1) No standalone & independent kitchen; 2) No independent water supply; 3) No hot water & separate bathing facilities; 4) No flush & separate toilet; 5) Briquettes, stoves, or conventional heating |
|  |  | Proportion of single-person households | The number of single-person households divided by the total number of households |
|  |  | Proportion of households with a female household head | The number of households with a female household head divided by the total number of households |
|  |  | Proportion of households not living in an apartment | The number of households with apartment housing divided by the total number of households |
|  |  | Proportion of people aged 65 years or over | The number of people aged 65 years or over divided by the total population |
|  |  | Proportion of people without a high school diploma | The number of people aged 30 to 64 years without a high school diploma divided by the total population in this age group |
|  |  | Proportion of divorced or widowed individuals | The number of people aged 15 years or over who are divorced or widowed divided by the total population in this age group |
| Medical index | Basic statistic database by region via Korean Statistical Information Service | Number of medical personnel per capita | The number of medical personnel (doctors, nurse practitioners, pharmacists, nurses, and nursing assistants) divided by the total population |
|  |  | Number of hospitals per capita | The number of hospitals (general hospital, hospital, clinic, nursing home, oriental hospital, and oriental clinic) divided by the total population |
|  |  | Number of hospital beds per capita | The number of hospital beds (general hospital, hospital, clinic, nursing home, oriental hospital, and oriental clinic) divided by the total population |

**Table S2.** Summary statistics of PM_2.5_ exposures and community-level variables.

|  | Time window | Mean | SD | Min | Q1 | Median | Q3 | Max |
| --- | --- | --- | --- | --- | --- | --- | --- | --- |
| PM_2.5_ (μg/m^3^) | Mov1 | 26.44 | 2.99 | 17.06 | 24.39 | 26.45 | 28.45 | 41.71 |
|  | Mov2 | 26.66 | 2.84 | 17.46 | 24.68 | 26.62 | 28.51 | 41.10 |
|  | Mov3 | 26.89 | 2.72 | 17.84 | 24.98 | 26.88 | 28.64 | 41.56 |
|  | Mov5 | 27.29 | 2.61 | 18.97 | 25.40 | 27.25 | 29.01 | 41.14 |
| Deprivation index | Mov1 | -3.68 | 5.17 | -14.95 | -7.34 | -4.96 | -0.93 | 15.10 |
|  | Mov2 | -3.71 | 5.10 | -14.95 | -7.24 | -5.03 | -1.08 | 15.11 |
|  | Mov3 | -3.74 | 5.05 | -14.95 | -7.23 | -5.09 | -1.21 | 15.32 |
|  | Mov5 | -3.80 | 4.97 | -14.87 | -7.21 | -5.12 | -1.41 | 15.48 |
| Medical index | Mov1 | 0.14 | 2.11 | -4.26 | -0.95 | -0.22 | 0.72 | 21.97 |
|  | Mov2 | 0.14 | 2.06 | -4.35 | -0.91 | -0.23 | 0.69 | 21.97 |
|  | Mov3 | 0.13 | 2.02 | -4.35 | -0.90 | -0.26 | 0.69 | 21.97 |
|  | Mov5 | 0.13 | 1.95 | -3.78 | -0.86 | -0.23 | 0.69 | 21.60 |
| NDVI | Mov1 | 0.57 | 0.14 | 0.22 | 0.46 | 0.59 | 0.68 | 0.85 |
|  | Mov2 | 0.56 | 0.13 | 0.22 | 0.46 | 0.58 | 0.67 | 0.85 |
|  | Mov3 | 0.56 | 0.13 | 0.22 | 0.46 | 0.58 | 0.67 | 0.84 |
|  | Mov5 | 0.56 | 0.13 | 0.21 | 0.46 | 0.57 | 0.66 | 0.83 |
| *Abbreviations:* SD, standard deviation; Min, minimum; Q1, first quartile; Q3, third quartile; Max, maximum; Mov1, 1-year moving average; Mov2, 2-year moving average; Mov3, 3-year moving average; Mov5, 5-year moving average; NDVI, normalized difference vegetation index. | | | | | | | | |

**Table S3.** Pearson's correlation coefficients between PM_2.5_ exposure and community level-variables.

|  | Mov1 PM_2.5_ | Mov2 PM_2.5_ | Mov3 PM_2.5_ | Mov5 PM_2.5_ | Mov5 NDVI | Mov5 Dpv | Mov5 Med |
| --- | --- | --- | --- | --- | --- | --- | --- |
| Mov1 PM_2.5_ | 1.00 | 0.96 | 0.94 | 0.89 | Not compared | | |
| Mov2 PM_2.5_ |  | 1.00 | 0.98 | 0.94 |  |  |  |
| Mov3 PM_2.5_ |  |  | 1.00 | 0.97 |  |  |  |
| Mov5 PM_2.5_ |  |  |  | 1.00 | -0.01 | -0.14 | -0.20 |
| Mov5 NDVI |  |  |  |  | 1.00 | 0.20 | -0.19 |
| Mov5 Dpv |  |  |  |  |  | 1.00 | 0.22 |
| Mov5 Med |  |  |  |  |  |  | 1.00 |
| *Abbreviations:* Mov1, 1-year moving average; Mov2, 2-year moving average; Mov3, 3-year moving average; Mov5, 5-year moving average; NDVI, normalized difference vegetation index; Dpv, deprivation index; Med, medical index. | | | | | | | |

**Table S4.** Hazard ratios (HRs) and 95% confidence intervals (CIs) of mortality associated with 10 μg/m^3^ increase in PM_2.5_ with additional adjustments.

| Cause of death | Adjustment | HR (95% CI) |
| --- | --- | --- |
| Non-accidental | Main model | 1.10 (1.01, 1.20) |
|  | Main model + NO_2_ | 1.07 (0.97, 1.17) |
|  | Main model + O_3_ | 1.09 (0.99, 1.19) |
|  | Main model + Temperature | 1.09 (0.99, 1.19) |
| Circulatory disease | Main model | 1.22 (1.01, 1.47) |
|  | Main model + NO_2_ | 1.24 (1.02, 1.50) |
|  | Main model + O_3_ | 1.23 (1.02, 1.48) |
|  | Main model + Temperature | 1.19 (0.99, 1.44) |
| Respiratory disease | Main model | 1.33 (1.05, 1.67) |
|  | Main model + NO_2_ | 1.37 (1.09, 1.74) |
|  | Main model + O_3_ | 1.33 (1.05, 1.68) |
|  | Main model + Temperature | 1.33 (1.05, 1.67) |
| *Note.* Main model is stratified by age and sex, and adjusted for income, smoking, alcohol use, physical activity, body mass index, medical index, deprivation index, normalized difference vegetation index, and indicator for province. | | |

**Table S5.** Hazard ratios (HRs) and 95% confidence intervals (CIs) of mortality associated with 10 μg/m^3^ increase in PM_2.5_, stratified by deprivation index, medical index, and NDVI (normalized difference vegetation index).

| Community-level variables | | Non-accidental | | Circulatory | | Respiratory | |
| --- | --- | --- | --- | --- | --- | --- | --- |
|  |  | HR (95% CI) | *P*_int._ | HR (95% CI) | *P*_int._ | HR (95% CI) | *P*_int._ |
| Deprivation index | |  |  |  |  |  |  |
|  | Low | 1.04 (0.86, 1.25) | - | 1.16 (0.79, 1.72) | - | 1.09 (0.66, 1.81) | - |
|  | Med | 1.13 (0.94, 1.34) | 0.74 | 1.41 (0.97, 2.04) | 0.11 | 1.60 (0.98, 2.60) | 0.47 |
|  | High | 1.14 (0.99, 1.33) | 0.72 | 1.16 (0.85, 1.57) | 0.67 | 1.66 (1.15, 2.41) | 0.61 |
| Medical index | |  |  |  |  |  |  |
|  | Low | 1.25 (1.08, 1.44) | - | 1.14 (0.84, 1.55) | - | 1.31 (0.88, 1.95) | - |
|  | Med | 1.04 (0.86, 1.26) | 0.19 | 1.19 (0.80, 1.77) | 0.47 | 1.56 (0.96, 2.53) | 0.55 |
|  | High | 0.99 (0.85, 1.17) | 0.03 | 1.27 (0.91, 1.76) | 0.89 | 1.37 (0.91, 2.09) | 0.89 |
| NDVI | |  |  |  |  |  |  |
|  | Low | 1.08 (0.88, 1.33) | - | 1.65 (1.09, 2.50) | - | 1.03 (0.61, 1.75) | - |
|  | Med | 1.11 (0.95, 1.30) | 0.29 | 1.27 (0.92, 1.76) | 0.33 | 1.75 (1.06, 2.87) | 0.53 |
|  | High | 1.11 (0.96, 1.29) | 0.13 | 1.06 (0.78, 1.46) | 0.07 | 1.66 (1.15, 2.38) | 0.78 |
| *Note.* *P*_int_ represents p-value for interaction term between PM_2.5_ and each community-level variable | | | | | | | |


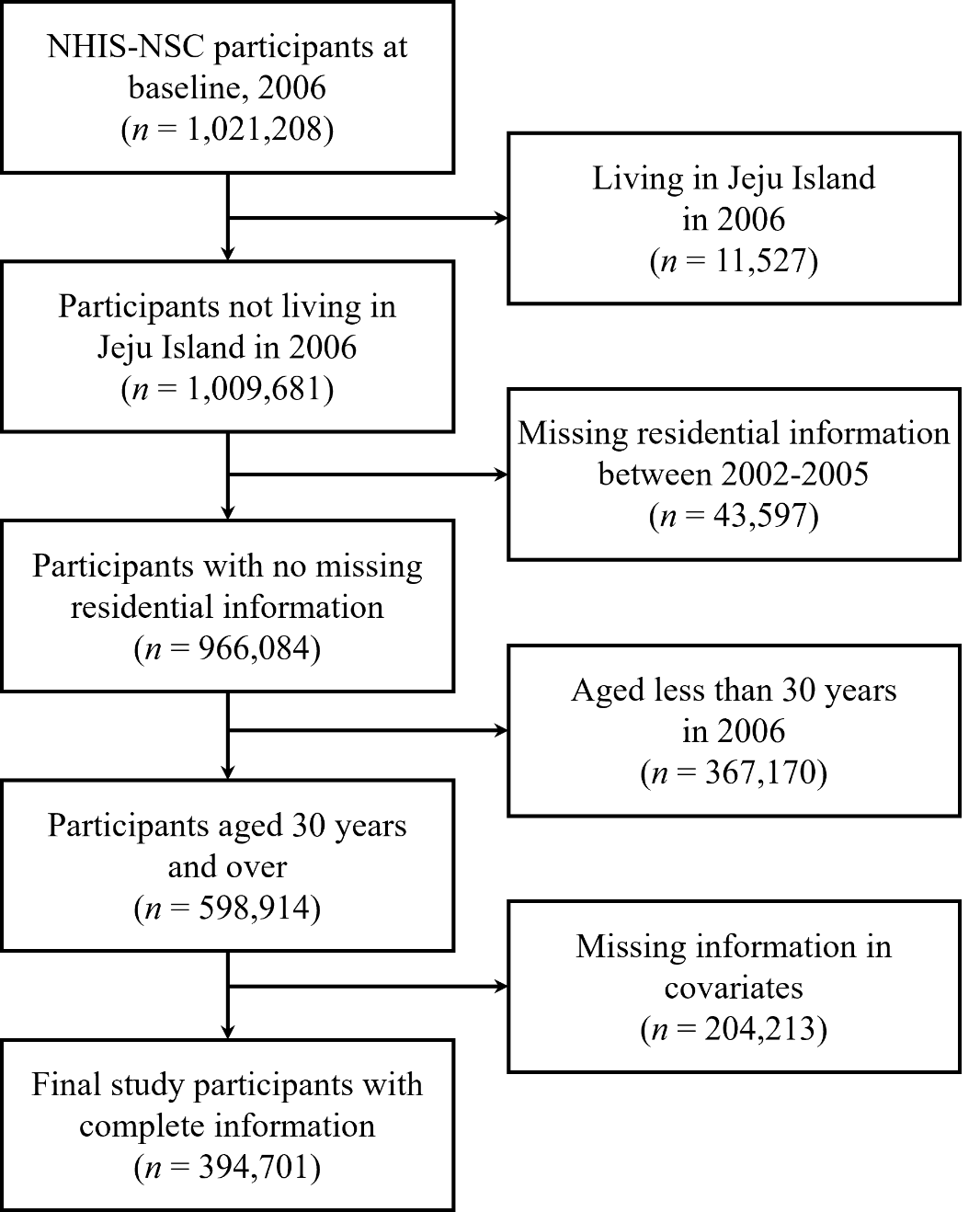


**Figure S1.** Flow chart of the selection process of the study participants


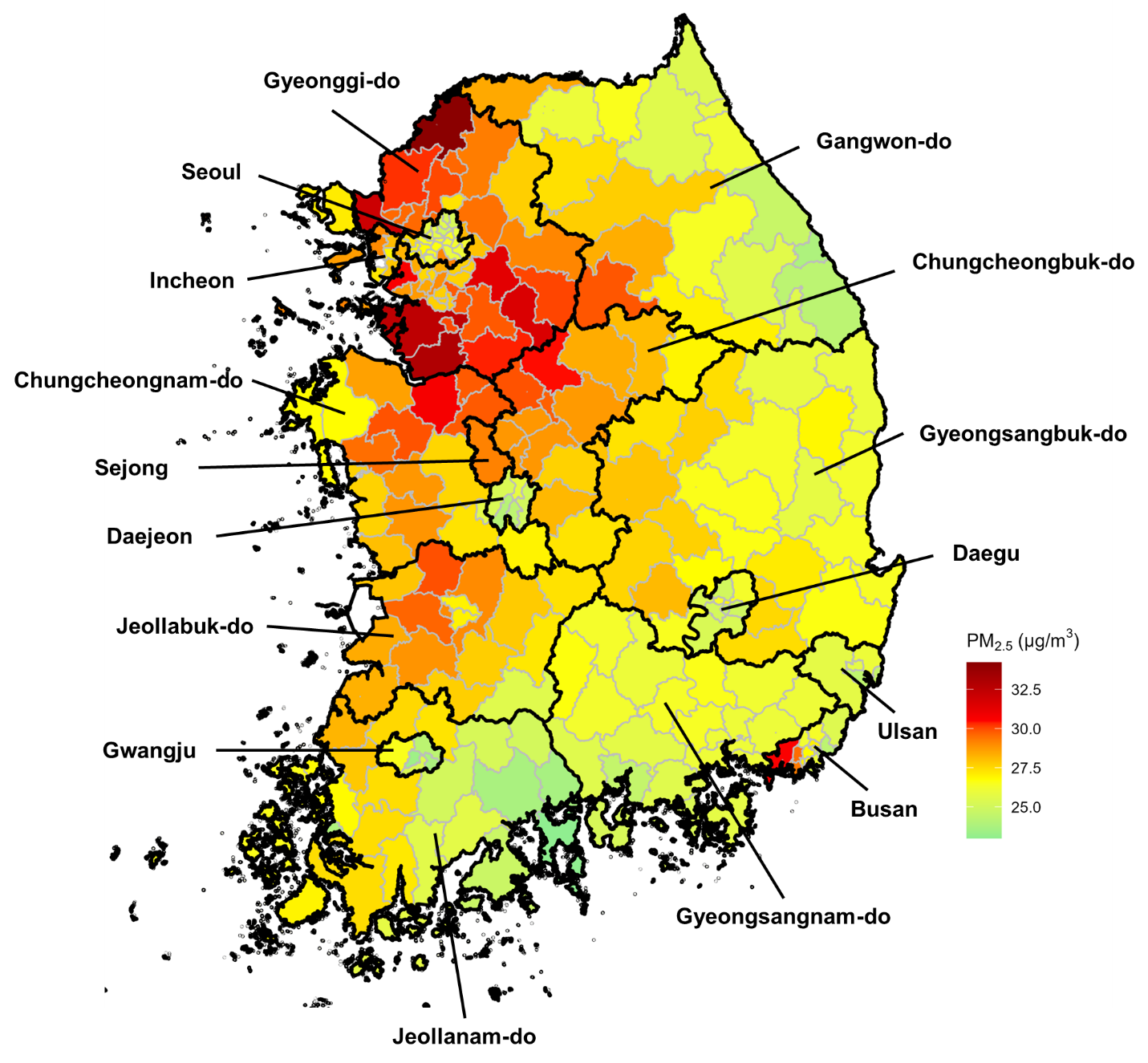


**Figure S2.** Map of predicted PM_2.5_ concentrations averaged at the district level
from 2002 to 2019 in South Korea.
*Note: Black line indicates provincial boundaries, and grey line indicates district boundaries.*


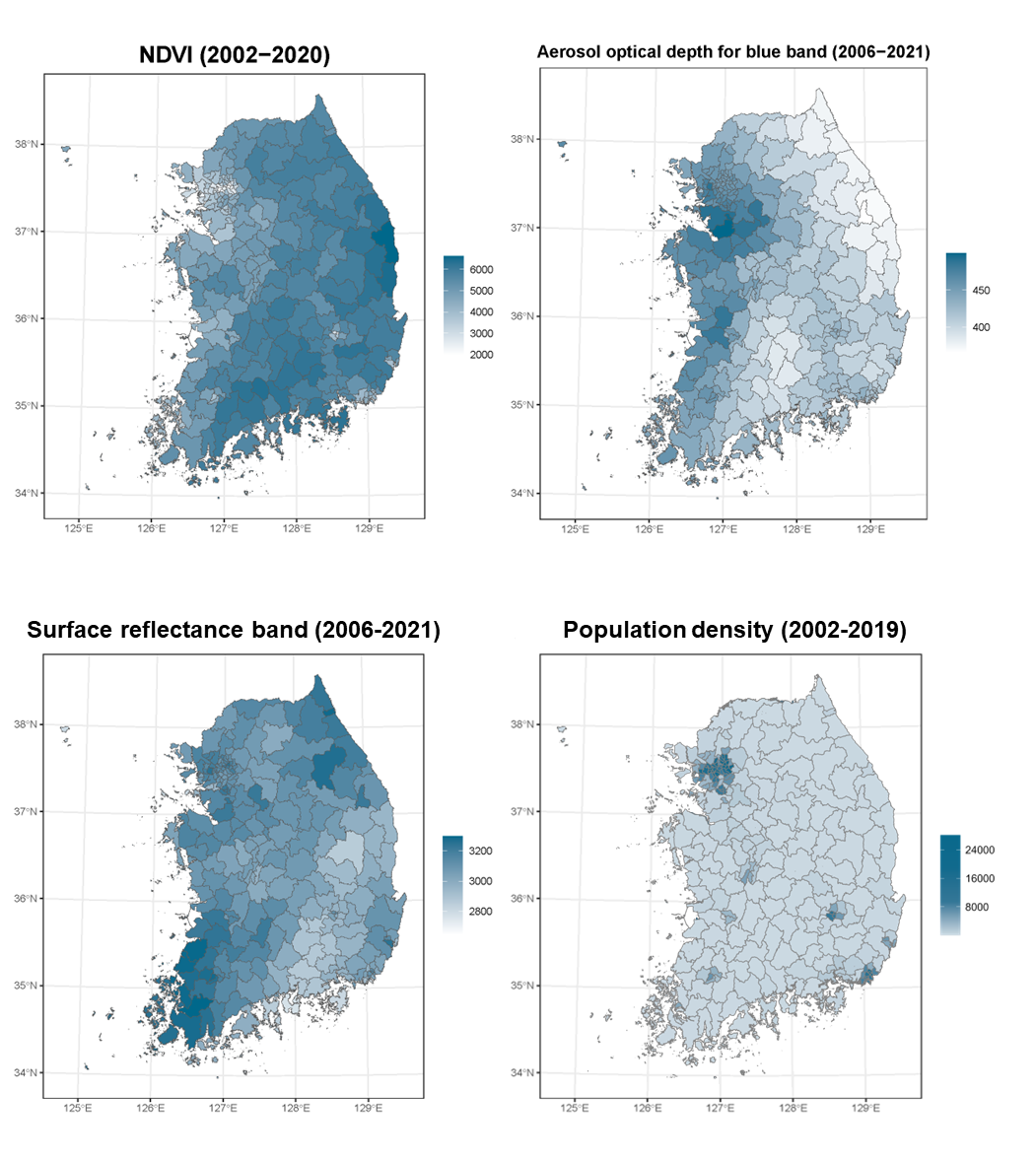


**Figure S3.** Maps of geospatial variables at the district level in South Korea.
*Note: The scale factor for NDVI and blue band AOD is 0.0001, the scale factor for surface reflectance band (620-670nm) is 0.001, and the unit for population density is population/km^2^.*
